# [18F]FDG-PET in a case of right temporal lobe variant of frontotemporal dementia

**DOI:** 10.1590/1980-57642018dn13-030013

**Published:** 2019

**Authors:** Marcelo Houat de Brito, Thiago Bezerra Moraes Teixeira, Poliana Fonseca Zampieri, Artur Martins Coutinho, Sonia Maria Dozzi Brucki

**Affiliations:** 1 University of São Paulo School of Medicine Hospital das Clínicas Department of Neurology São PauloSP Brazil Hospital das Clínicas, University of São Paulo School of Medicine, Department of Neurology, São Paulo, SP, Brazil.; 2 University of Sao Paulo School of Medicine Hospital das Clínicas Nuclear Medicine Center São PauloSP Brazil Hospital das Clínicas, University of Sao Paulo School of Medicine, Nuclear Medicine Center, Department of Radiology, São Paulo, SP, Brazil.

**Keywords:** positron-emission tomography, fluorodeoxyglucose F18, frontotemporal dementia, tomografia por emissão de pósitrons, fluordeoxiglicose F18, demência frontotemporal

A 61-year-old woman presented with a 4-year history of slowly progressive amnestic and topographical disorientation symptoms, followed by an early onset of apathy, hyperorality, ritualistic behaviors, prosopagnosia, and phonagnosia. Due to diagnostic uncertainty between Frontotemporal Dementia (FTD) and Alzheimer's Disease (AD) pathology, CSF biomarker analysis and brain [18F]FDG-PET were ordered. The first proved negative for AD profile, and the latter demonstrated marked hypometabolism in the right temporal and frontal lobes, areas that also had asymmetric atrophy on brain MRI. These findings, together with the clinical picture, were compatible with the hypothesis of a right temporal lobe variant of FTD.^1^^,^^2^

**Figure:**
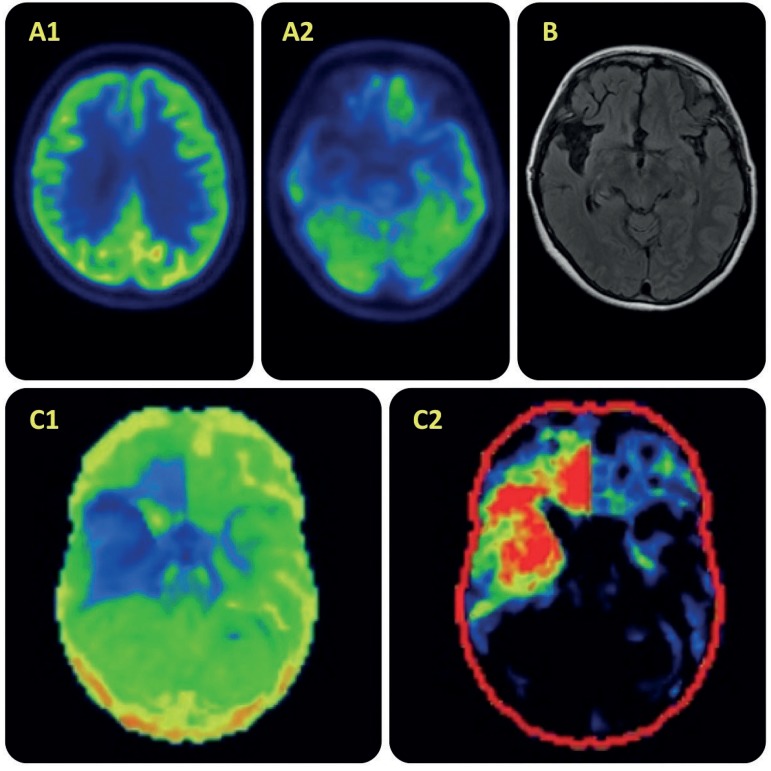
Upper row - A1, A2: [18F]FDG-PET transaxial images showing prominent hypometabolism in the right temporal lobe and, to a lesser degree, in the right frontal and parietal lobes, particularly orbitofrontal areas. B - transaxial T2 FLAIR MRI image showing selective right temporal atrophy. Lower row - C1: a 3D-SSP reconstruction (inferior view) showing prominent hypometabolism in the right temporal lobe and right orbitofrontal cortex. C2 - 3D-SSP projection of comparison with a group of healthy age-matched individuals. Red areas indicate areas of lower metabolism than expected 423x423mm (72 x 72 DPI).
